# Supplementary material for: Atg18 oligomer organization in assembled tubes and on lipid membrane scaffolds
Source: Nat Commun. 2023 Dec 6;14:8086. doi: 10.1038/s41467-023-43460-3 (PMC10700546; doi:10.1038/s41467-023-43460-3)
Supplement: Supplementary file 1 — Supplementary Information [file 41467_2023_43460_MOESM1_ESM.pdf]

# Supplementary information

## Atg18 oligomer organization in assembled tubes and on lipid membrane scaffolds

Daniel Mann, Simon A. Fromm et al.

## Supplementary Figures

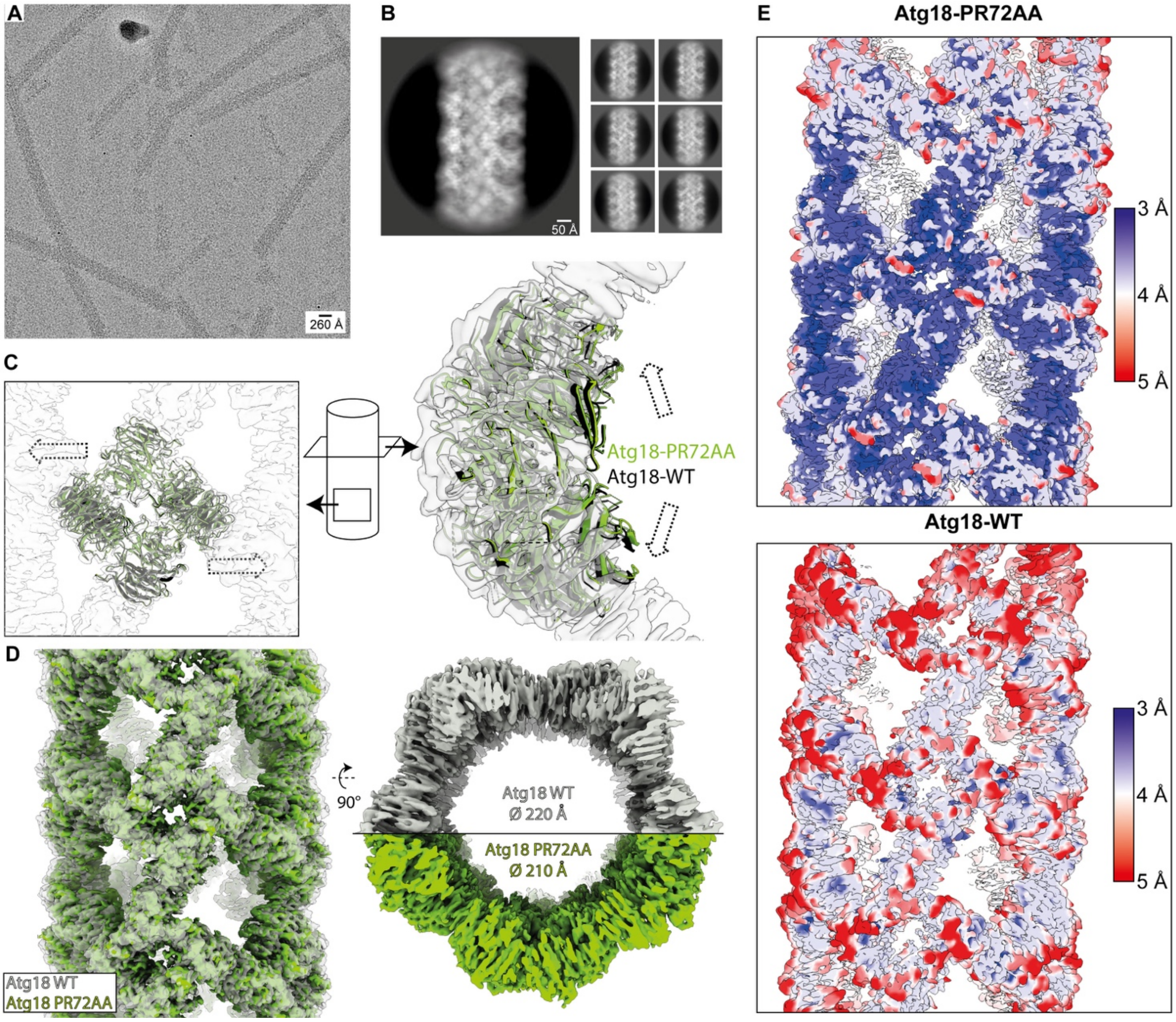


***Suppl. Fig. 1: Helical structure of Atg18 in comparison with Atg18-PR72AA.***

*(A) Typical electron cryo-micrograph of Atg18-wildtype(WT) tubes. (B) Representative 2D classes of Atg18-WT. (C) Comparison of molecular models of Atg18-WT (black) and Atg18-PR72AA (green) with the Atg18-WT cryo-EM map superimposed in transparent grey. (D) 3D maps of Atg18-WT and Atg18-PR72AA cryo-EM density maps superimposed. Comparison of respective cryo-EM densities reveals slightly wider diameter of Atg18-WT compared with Atg18-PR72AA. Note that the data were acquired on the same microscope with the same pixel size. (E) Local resolution mapped onto the isosurface of Atg18-PR72AA and Atg18-WT filaments determined by FDR-FSC at 1% FDR.*

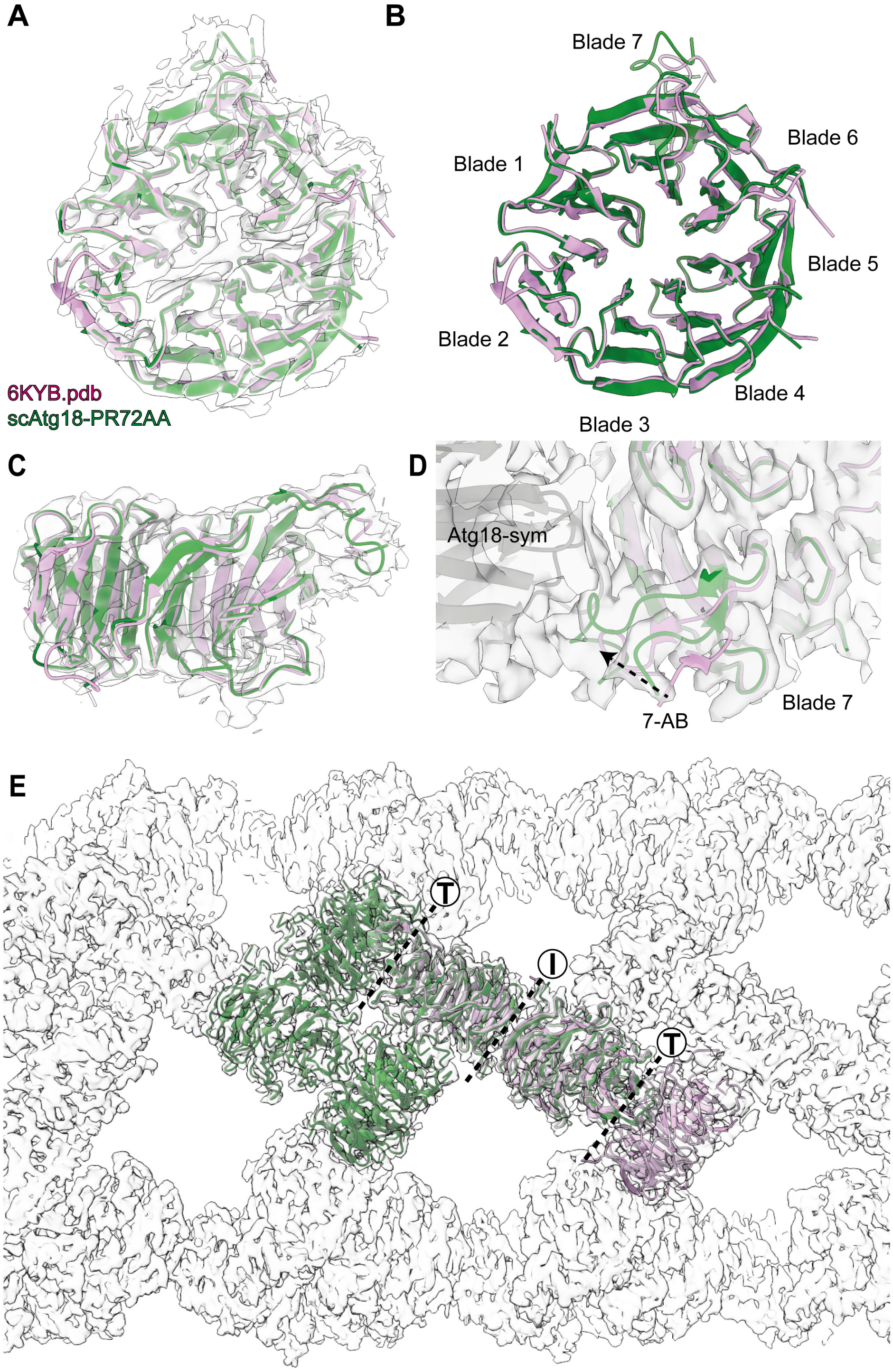


***Suppl. Fig. 2: Comparison of Atg18-PR72AA cryo-EM tube structure with X-ray structure of Atg18 (PDB-ID 6KYB).***

*(A) The overall structure is very similar between Atg18-PR72AA (green) and Atg18 (PDB-ID 6KYB [https://doi.org/10.2210/pdb6KYB/pdb]) (magenta) both of which match the experimental cryo-EM density (grey). Superposition in (B) top and (C) side view shows minor differences in the backbone of the 7AB loop in blade 7. (D) Close up reveals that loop 7AB is bound more closely to the symmetry neighbor in the tubular assembly (grey). (E) 6KYB PDB structure (magenta) contains the same I and T-interfaces as the determined tubular cryo-EM structure (green). Cryo-EM density of the helical tube is displayed in grey. 6KYB PDB structure was structurally aligned to the Atg18-PR72AA atomic model using the matchmaker program in ChimeraX.*

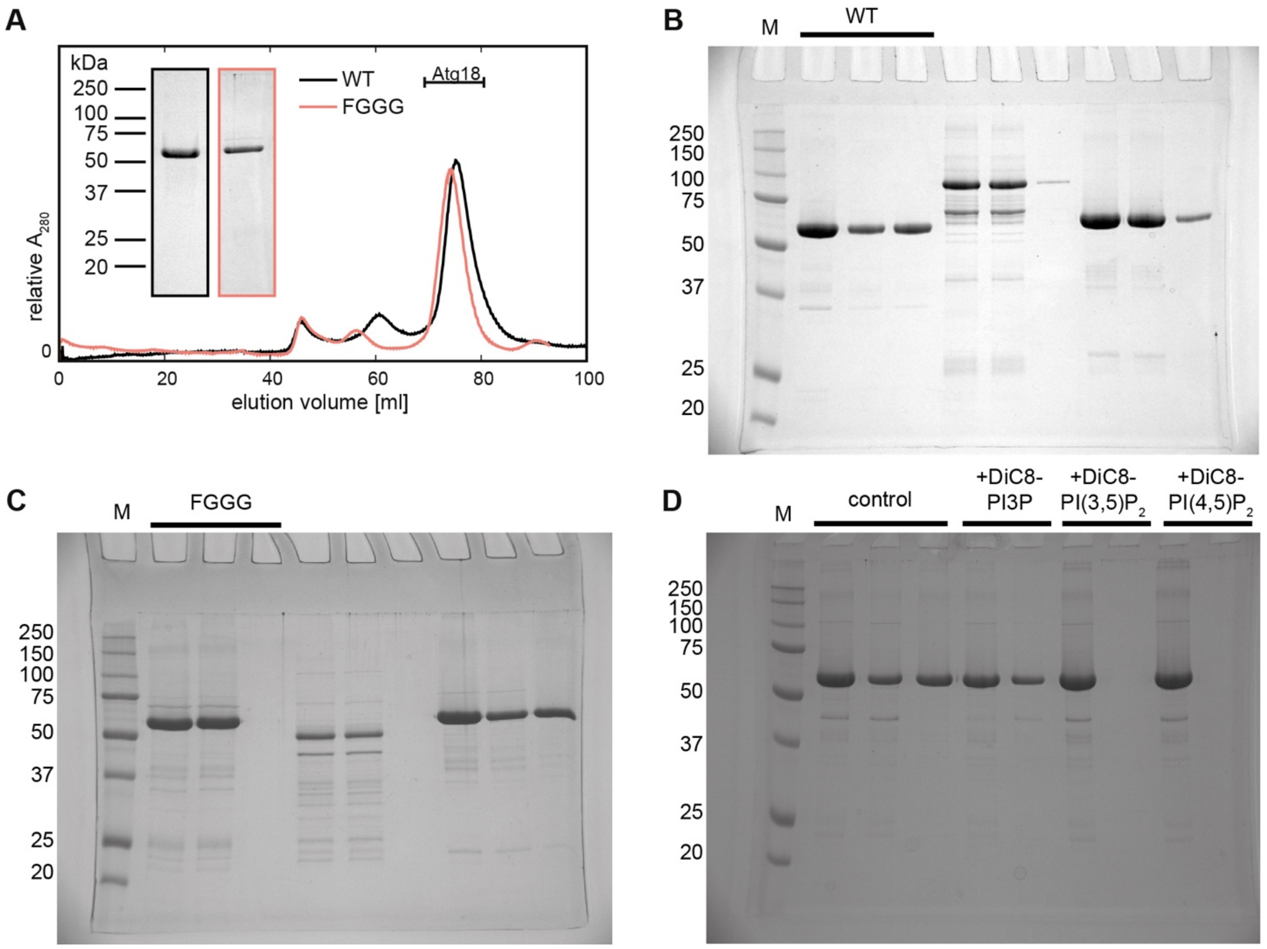


***Suppl. Fig. 3. Gel filtration profiles and pelletation assay of Atg18 and Atg18-FGGG.***

*(A) Gel filtration profile of Atg18-wildtype(WT) (black) and Atg18-FGGG (rose). Uncropped and unedited Coomassie-stained SDS-PAGE gels of pelletation assay used for preparing Fig. 3: (B) Atg18-WT, (C) FGGG mutant, and (D) Atg18 incubated with water (control), soluble DiC8-PI3P, DiC8-PI(3,5)$P_2$ and DiC8-PI(4,5)$P_2$ as indicated on top of the lanes. Molecular weight standards are indicated on the left next to the precision plus standard marker M lanes. Source data are provided as a Source Data file.*

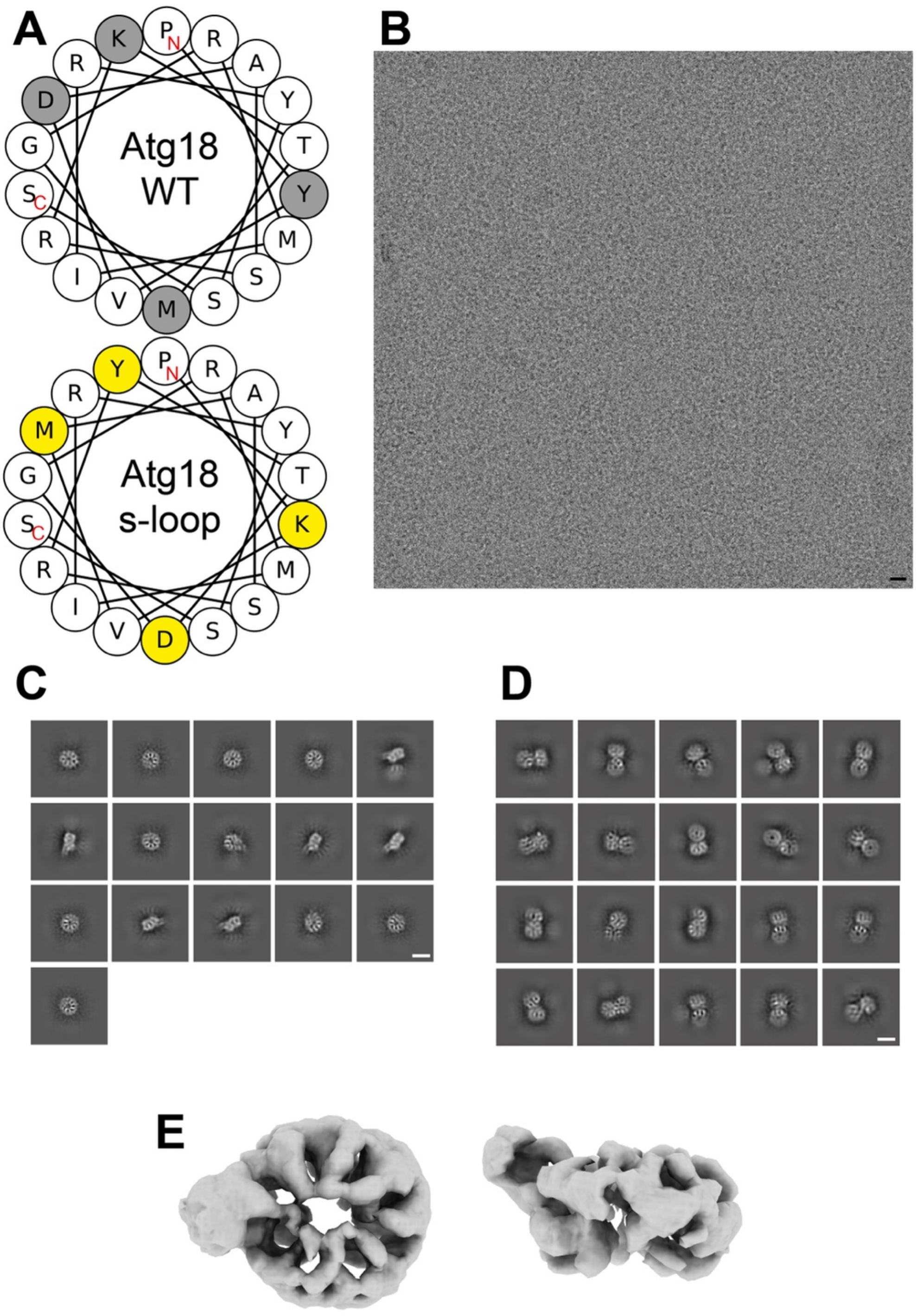


***Suppl. Fig. 4. Atg18 scrambled-loop characterization***

*(A) Helical wheel plot of the scrambled 6CD loop of Atg18 (Atg18 s-loop) (top) and including the point mutations K367Y, M369, Y373K and D375M (bottom). (B) Typical micrograph of Atg18 s-loop in vitreous ice (Titan Krios G4 300kV, Falcon IV detector, 60 $e^{-}$/Å$^{2}$ total fluence, nominal defocus -2 µm, scale bar 20 nm). (C) 2D classes of Atg18 s-loop monomers with visible β-propeller fold. (D) 2D classes of Atg18 s-loop dimers with visible β-propeller fold. (E) Initial 3D model of Atg18 s-loop confirms that the protein is correctly folded and has a comparable globular shape to the wildtype protein. Note that the flexible 6CD loop is not visible in the density.*

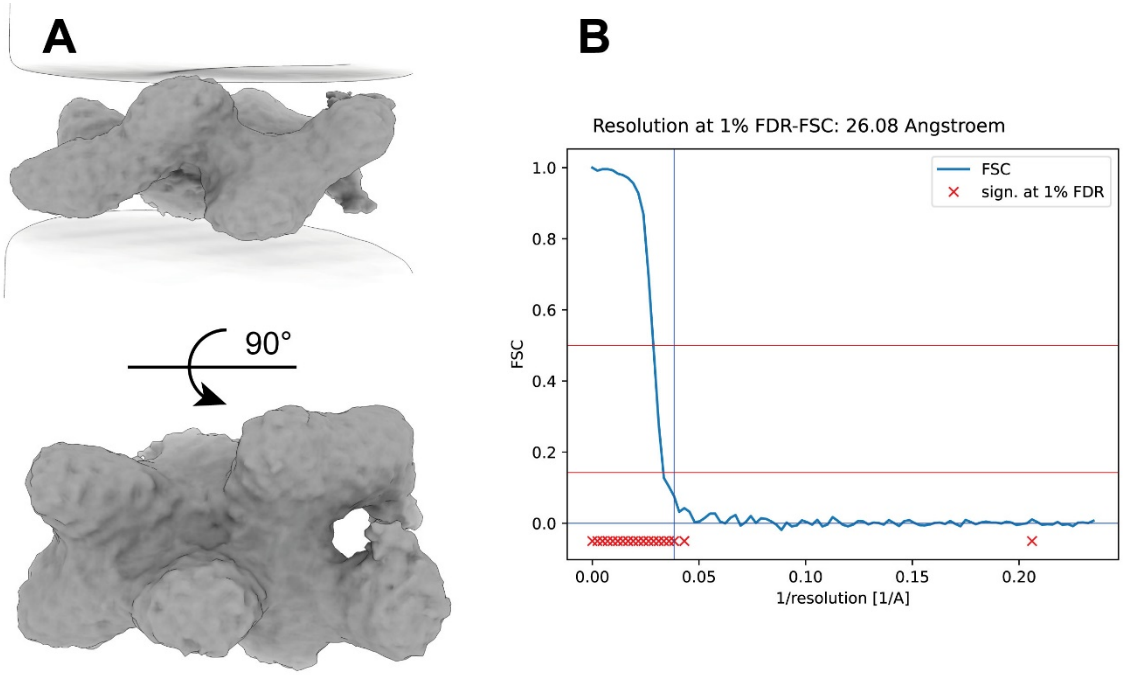


***Suppl. Fig. 5. Subtomogram average structure of membrane-bound Atg18.***

*(A) Subtomogram average density of membrane-bound Atg18 and (B) the corresponding resolution determination by FSC at 1% FDR.*

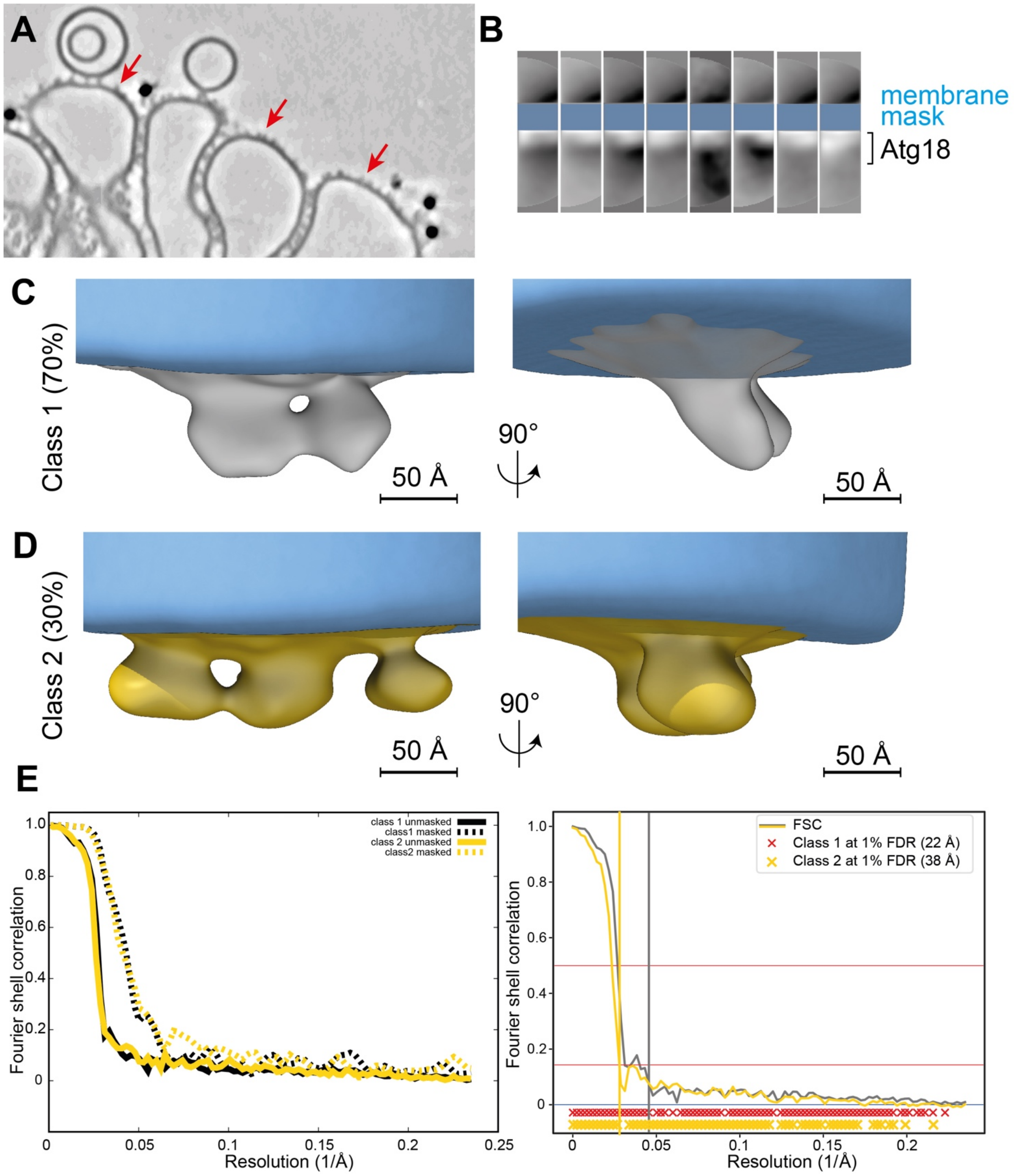


***Suppl. Fig. 6. Subtomogram averaging of Atg18 coating single phospholipid bilayers.***

*(A) Representative tomogram area of LUVs (95%DOPC 5% PIP3,5P$_2$) with Atg18 coats. (B) Selected rotationally averaged 2D classes. (C-D) 3D classes of membrane-bound Atg18 reveals that the majority of particles are bound to the membrane at an angle of approximately 45°. (E) Fourier shell correlation of subtomogram average indicating a nominal resolution of 28 Å (FSC=0.143) for both classes. FDR-FSC at 1% indicates slightly lower resolution for class 2 (38 Å) than for class 1 (22 Å).*

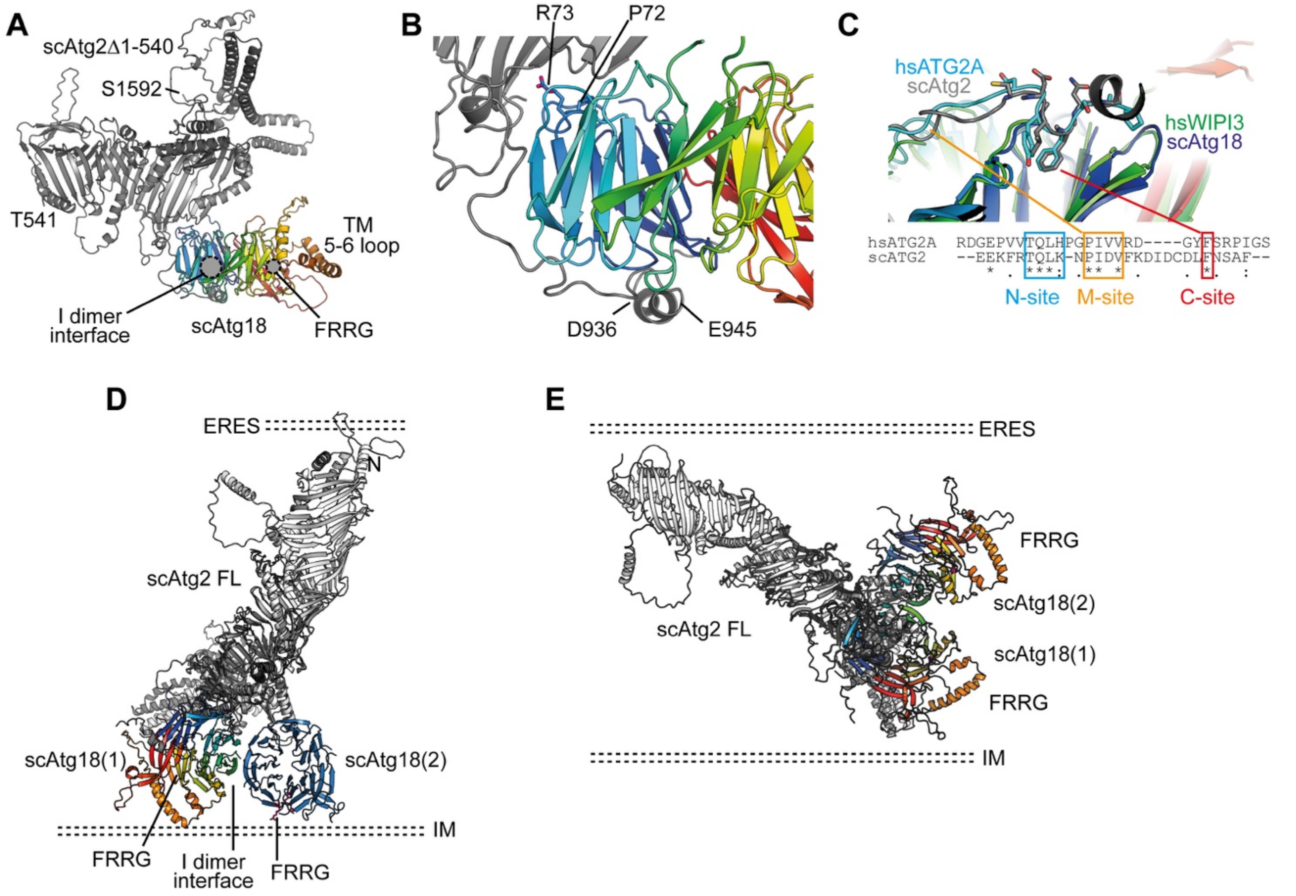


***Suppl. Fig. 7. Alphafold2 prediction of the Atg18-Atg2 interface.***

*(A) Alphafold2 complex prediction of Atg18 (rainbow) and C-terminal Atg2 (grey). (B) Detailed predicted binding interface with Atg18-P72-R73 (rainbow) and Atg2-D936-E945 (grey) highlighted in the two binding sites. (C) Detailed comparison of scAtg2-scAtg18 binding and hsAtg2A-hsWipi3 binding with conserved N-site, M-site and C-site. (D-E) Models of 2xAtg18-Atg2 membrane binding in autophagy based on Atg18 oligomerization site I and the Alphafold2 prediction (IM=isolation membrane; ERES=Endoplasmic reticulum exit site).*

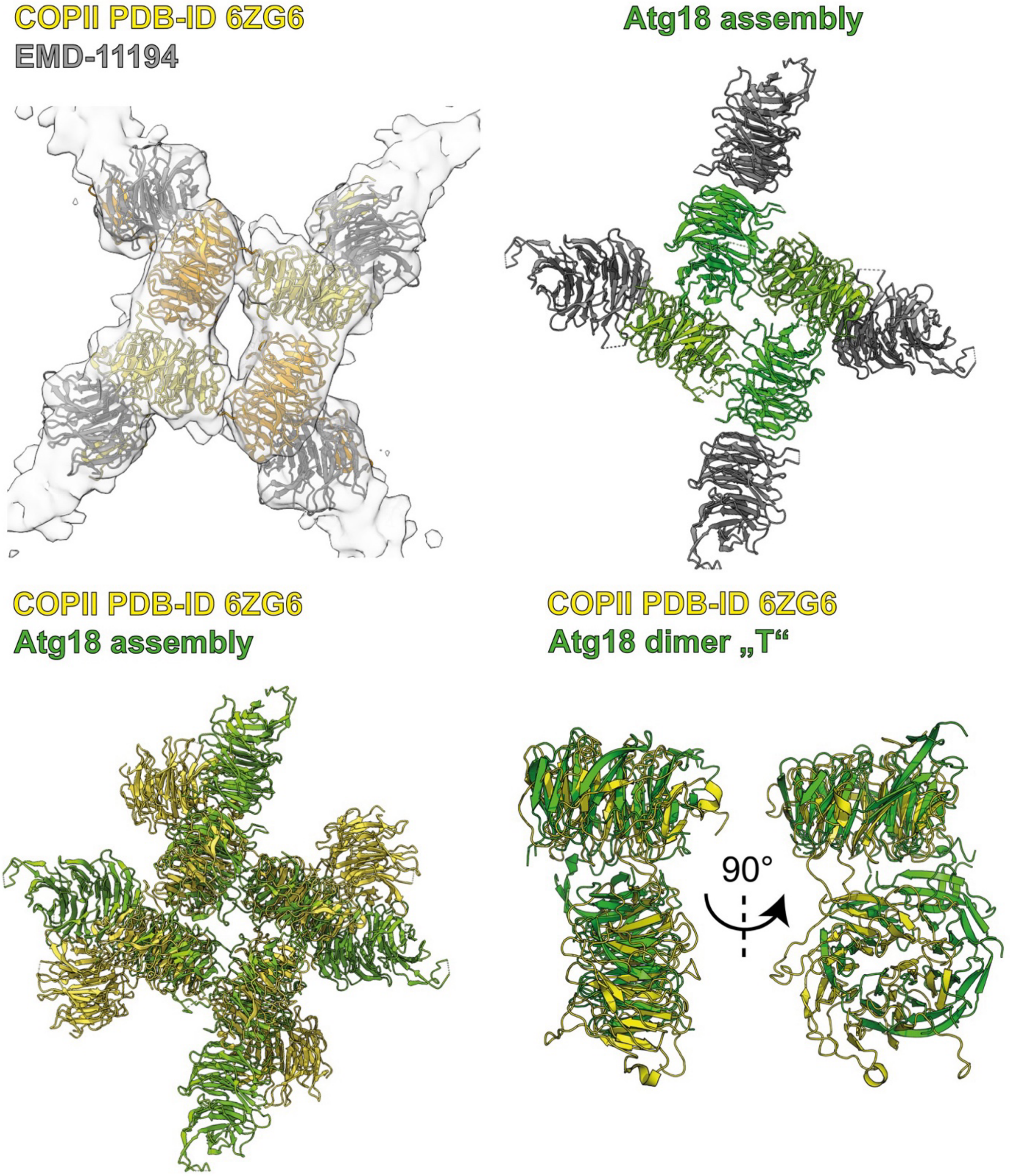


***Suppl. Fig. 8. Structural comparison of outer coat COPII architecture with Atg18 tubular assembly.***

*Top: Side views of lozenge pattern for COPII/Sec13-Sec31 (PDB ID 6ZG6 [https://doi.org/10.2210/pdb6ZG6/pdb]) with corresponding cryo-EM density (EMD-11194 [https://www.ebi.ac.uk/pdbe/entry/emdb/EMD-11194]) (left, yellow) and Atg18 (right, green). Bottom left: Side view of superimposed diamond-shaped COPII/Sec13-Sec31 (yellow) and Atg18 (green) atomic models. Bottom right: T-interface superposition of atomic models of COPII/Sec13-Sec31 (yellow) and Atg18 (green).*

***Supp. Movie 1: Denoised tomogram (related to Fig. 5)***
