# Supplementary material for: Atg18 oligomer organization in assembled tubes and on lipid membrane scaffolds
Source: Nat Commun. 2023 Dec 6;14:8086. doi: 10.1038/s41467-023-43460-3 (PMC10700546; doi:10.1038/s41467-023-43460-3)
Supplement: Supplementary file 3 — Description of additional supplementary files [file 41467_2023_43460_MOESM3_ESM.pdf]

Description of additional supplementary files

Supplementary Movie 1: Denoised tomogram from Fig. 5
